# Relation Between the Number of Peaks and the Number of Reciprocal Sign Epistatic Interactions

**DOI:** 10.1007/s11538-022-01029-z

**Published:** 2022-06-17

**Authors:** Raimundo Saona, Fyodor A. Kondrashov, Ksenia A. Khudiakova

**Affiliations:** Institute of Science and Technology Austria, Am Campus 1, 3400 Klosterneuburg, Lower Austria Austria

**Keywords:** Fitness landscapes, Multiple peaks, Morse theory, Reciprocal sign epistasis

## Abstract

Empirical essays of fitness landscapes suggest that they may be rugged, that is having multiple fitness peaks. Such fitness landscapes, those that have multiple peaks, necessarily have special local structures, called reciprocal sign epistasis (Poelwijk et al. in J Theor Biol 272:141–144, 2011). Here, we investigate the quantitative relationship between the number of fitness peaks and the number of reciprocal sign epistatic interactions. Previously, it has been shown (Poelwijk et al. in J Theor Biol 272:141–144, 2011) that pairwise reciprocal sign epistasis is a necessary but not sufficient condition for the existence of multiple peaks. Applying discrete Morse theory, which to our knowledge has never been used in this context, we extend this result by giving the minimal number of reciprocal sign epistatic interactions required to create a given number of peaks

## Introduction

The fitness landscape is the relationship between genotypes and their fitness. Availability of high throughput methods and next-generation sequencing started to experimentally characterize aspects of different fitness landscapes. Due to the enormity of the underlying genotype space (Maynard Smith 1970; Wright 1932), the experimental approaches are limited to assaying the fitness of: (a) closely related genotypes (Melamed et al. 2013; Romero and Arnold 2009; Sarkisyan et al. 2016; de Visser and Krug 2014); or (b), very restricted genotype spaces such as the interaction of a small number of protein sites (Kuo et al. 2020; Pokusaeva et al. 2019; Wittmann et al. 2021). Nevertheless, the number of assayed genotypes in a single landscape is becoming larger in recent studies (Bryant et al. 2021; Russ et al. 2020) and it appears that the experimental characterization of a sufficiently large fitness landscape with multiple fitness peaks may be attainable within the next decade. The possibility of characterizing multiple fitness peaks will always be restricted by the boundaries of the studied genotype space, thus what appears to be two unconnected fitness peaks may be found along the same fitness ridge when a larger section of the genotype space is analyzed (Whitlock et al. 1995). Therefore, there is a need for the development of computational methods (Alley et al. 2019; Bryant et al. 2021; Russ et al. 2020; Biswas et al. 2021; Wittmann et al. 2021) and theory (Zhou and McCandlish 2020) that can improve the description of experimental fitness landscape datasets, such as obtaining an estimate of the number of isolated peaks. Here, we use Morse theory to calculate the minimal number of reciprocal epistatic interactions for a given number of peaks on a landscape.

Epistasis is the interaction of allele states of the genotype, which shapes the fitness landscape. When the impact of allele states on fitness is independent of each other, there is no epistasis and the resulting fitness landscape is smooth and has a single peak. Epistasis can lead to a more rugged fitness landscape and decrease the number of paths of high fitness between genotypes. Epistasis that makes the impact of an allele state on fitness stronger or weaker is called *magnitude epistasis*. On the other hand, epistasis that causes the contribution of an allele state on fitness to change its sign (e.g., a beneficial mutation becomes deleterious) is called *sign epistasis*  (Weinreich et al. 2005). When the two allele states at different loci change the sign of their respective contribution to fitness, then this interaction is called *reciprocal sign epistasis*.

**Fig. 1:**
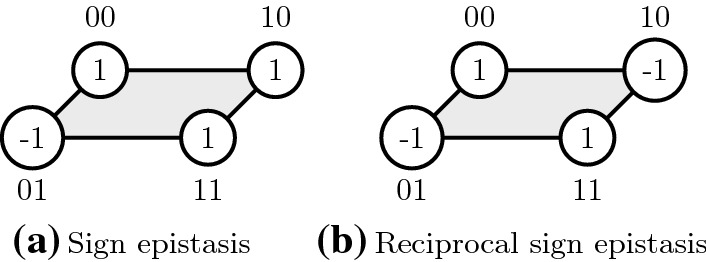
Types of sign epistasis. Vertices represent genotypes (the sequence is outside the vertex and the fitness inside); edges are present between sequences at mutation distance one; and filled faces represent sign epistatic interactions

In a simple example of this principle, in a two-loci two-allele model, there are four genotypes, 00, 01, 01 and 11. The following landscape is shaped by sign epistasis when genotypes 00, 01, 10 and 11 have fitnesses of 1,−1,1 and 1, respectively. Reciprocal sign epistasis (RSE) is present when the fitnesses of 00, 01, 10 and 11 genotypes are 1,−1,−1 and 1, respectively. See Fig. 1 for an illustration.

Of course, the effect of an allele state can depend on more than just one other locus, or site, in the genome. When allele states in different loci impact each other, then the epistasis is higher-order. Higher-order epistasis is found frequently in the characterized fitness landscapes (Weinreich et al. 2013), and it is clear that it has important evolutionary consequences  (Canale et al. 2018; Crona et al. 2019; Kondrashov and Kondrashov 2001; de Visser and Krug 2014). However, models that allow studying such epistasis are at an early stage of their development (Crona et al. 2013; Crona 2020), see also Crona et al. 2021 (preprint).

The evolutionary consequences of epistasis may be especially important when it leads to multiple local peaks. In that case, a population can get stuck on a suboptimal peak, decreasing the ability of evolution to find an optimal solution.

Using a combinatorial argument, Poelwijk et al. (2011) showed the following qualitative property: reciprocal sign epistasis is necessary for the existence of multiple peaks. In contrast, using Morse theory, we derive a more quantitative description of this relationship. This work might be the first formal use of Morse theory to study fitness landscapes.

## Outline of the Method

Morse theory studies the properties of some discrete structures (such as graphs) and special functions defined on them. In particular, the strong Morse inequality relates topological characteristics of a structure with the number of critical points of any function defined on it. Therefore, to use Morse Theory, we define a discrete structure that highlights reciprocal sign epistatic interactions and a function based on the given fitness landscape.

The discrete structure is a graph: vertices are binary sequences (genotypes) and edges connect those genotypes within one-mutation distances. Moreover, we include edges between those vertices that are separated by a reciprocal sign epistatic interaction.

In the case of graphs, the only requirement for (Morse) functions is to assign a number to both nodes and edges. Naturally, the value on the vertices corresponds to the genotype’s fitness. On the other hand, the value on the edges is tailored for Theorem 1.

### Theorem 1

(Quantification of epistatic interactions) Let genotypes be encoded as binary sequences. Consider a fitness landscape, i.e., a function that assigns a number to each genotype, with no strictly neutral mutations. Then,

$$\begin{aligned} \#\text { RSE instances }\ge \# \text { peaks }- 1 \,. \end{aligned}$$

Because we model genotypes as binary sequences, the sequence space is a hypercube. Also, we only consider fitness landscapes with no strictly neutral mutation, i.e., all direct neighbors of a vertex must have a different value than this vertex. These two assumptions allow us to unambiguously define RSE instances.

## Informal Proof

Let us first briefly explain the combinatorial argument used in Poelwijk et al. (2011) to show the following qualitative property: reciprocal sign epistasis is a necessary condition for the existence of multiple peaks. The main idea is that between any two peaks (i.e., genotypes with locally maximal fitness), there must be a path consisting of single mutations connecting them. In particular, if the path is chosen well, the minimum fitness along this path is part of a RSE instance. Theorem 1 is the corresponding quantitative version of this statement. In particular, if there are three peaks, we conclude that there is not only one instance of RSE in the fitness landscape, but there must exist at least two of them.

Intuitively, our result is explained by induction over the number of peaks as follows. In the base case, already explored in Poelwijk et al. (2011), there are only two peaks. For the inductive case, consider a fitness landscape and introduce a new peak to it. This new peak must be connected to all previous peaks through some reciprocal sign epistatic interaction. The question is if any of these interactions was not there before. We show that a new peak must introduce at least one more such interaction. To make this last step in the proof formal, we use discrete Morse theory.

**Fig. 2:**
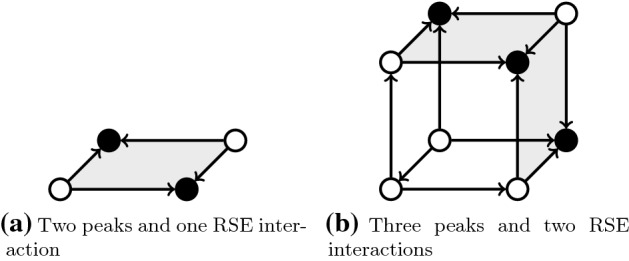
Introducing a peak introduces a RSE interaction. Vertices represent genotypes; arrows represent fitness increments; filled vertices represent peaks; and filled faces represent reciprocal sign epistatic interactions

If we allow to introduce a peak together with a new dimension, it is easy to illustrate the induction. For the case of two dimensions, we would introduce a third dimension together with a new peak. See Fig. 2b for a representation.

## Formal Proof

The strong Morse inequality is a general tool that relates characteristics of a space with properties of special functions defined on it. To motivate subsequent definitions, let us present the original statement (Forman 1998, Corollary 3.6, page 107) applied to graphs (instead of more general discrete structures).

### Theorem 2

(Strong Morse inequality) Consider a graph $G = (V, E)$ and a function $f: V \cup E \rightarrow \mathbb {R}$. Let $b_0$ and $b_1$ denote the first two Betti numbers of the graph *G* and let $m_0$ and $m_1$ denote the number of critical nodes and edges of *f*. Then, we have that

$$\begin{aligned} m_1 - m_0 \ge b_1 - b_0 \,. \end{aligned}$$

To use this result, we must define the following terms: Betti numbers and critical nodes and edges. But before we do that, note that if the number of RSE instances can be represented as a structural property of a graph (inside $b_1 - b_0$), and the number of peaks can be encoded in a function (inside $m_1 - m_0$), then this inequality allows us to quantify the necessary condition for the existence of multiple peaks.

We introduce all the necessary concepts before explaining the proof step by step.

### Necessary Definitions

In this section, we introduce the terms used in Theorem 2 (*Betti numbers*, *critical nodes* and *critical edges*), as well as *RSE instances*. All definitions coincide with those given in the general literature.

#### Definition 1

*(Betti numbers)* Let $G = (V, E)$ be a graph. The zeroth Betti number ($b_0$) is the number of connected components in *G*. The first Betti number ($b_1$) equals $|E |+ b_0 - |V |$, usually called cyclomatic number.

#### Remark 1

*(Betti numbers in connected graphs)* Let $G = (V, E)$ be a connected graph. Then, $b_0 = 1$ and $b_1 = |E |+ 1 - |V |$. Since *G* is connected, $|E |\ge |V |- 1$, therefore $b_1 \ge 0$.

#### Definition 2

*(Critical nodes and edges)* Let $G = (V, E)$ be a graph and $f: V \cup E \rightarrow \mathbb {R}$ a function. We say that a vertex $v \in V$ is *critical* if, for all edges *e* containing *v*, we have that $f(e) > f(v)$. We say that an edge $e = \{u, v\} \in E$ is *critical* if $f(e) > \max \{ f(u), f(v) \}$. We denote $m_0$ the number of critical vertices and $m_1$ the number of critical edges.

#### Definition 3

*(RSE instance)* Consider a fitness landscape represented by $W: \{0, 1\}^n \rightarrow \mathbb {R}$, where *n* is the length of the genotype. An *instance of RSE* is a collection of four different sequences $s_1, s_2, s_3, s_4 \in \{0, 1\}^n$ such that both sequences $s_1$ and $s_4$ are one single mutation away from $s_2$ and $s_3$ and it holds that

$$\begin{aligned} \max (W(s_2), W(s_3)) < \min (W(s_1), W(s_4)) \,. \end{aligned}$$

### Proof

#### Proof of Theorem 1

Let a fitness landscape be represented by a function $W:\{0, 1\}^n \rightarrow \mathbb {R}$, where *n* is the length of the genotype. Our proof consists of the following steps:

1. Define a graph.
2. Show that this graph is connected.
3. Define a function on the graph.
4. Apply the strong Morse inequality.

During the proof, we will instantiate our constructions in the following example.

#### Example 1

*(Fitness landscape in a cube)* Consider $n = 3$ and the fitness function $w :\{0, 1\}^n \rightarrow \mathbb {R}$ given as follows.

$$\begin{aligned} 000 \mapsto 0 \\ 100 \mapsto 3 \\ 010 \mapsto 1 \\ 001 \mapsto 3 \\ 110 \mapsto 2 \\ 101 \mapsto 2 \\ 111 \mapsto 3 \\ 101 \mapsto 2 \\ 011 \mapsto 2 \end{aligned}$$

**Fig. 3:**
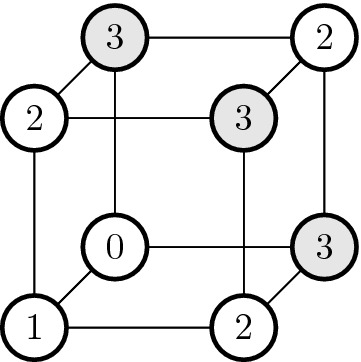
Fitness landscape in a cube. Vertices represent genotypes; edges connect genotypes at one-mutation distance; filled vertices represent peaks; and fitness is indicated in each vertex

A representation is given in Fig. 3.

***Definition of the graph***

Consider a graph $G = (V, E)$. Let $V :=\{0, 1\}^d$. Let the set of edges $E :=E_1 \cup E_2$ be defined in two steps: $E_1$ and $E_2$ have edges involving only sequences at Hamming distance one and two, respectively. The set $E_1$ contains only edges connecting a sequence with one of its fittest beneficial mutations, if one exists (peaks have no beneficial mutation). Formally,

$$\begin{aligned} E_1 \subseteq \{ \{u, v\} : d(u, v) = 1, W(u) < W(v) = \max \{ W(v') : d(u, v') = 1 \} \} \,, \end{aligned}$$

where *d* denotes the Hamming distance.

On the other hand, $E_2$ contains edges that connect the two highest points separated by instance of RSE. Formally,

$$\begin{aligned} E_2:= & {} \{ \{u, v\} : d(u, v) = 2, \forall y \in V \quad d(u, y) = 1 \wedge d(v, y) = 1 \Rightarrow W(y)\\&< W(u) \wedge W(y) < W(v) \} \,. \end{aligned}$$

**Fig. 4:**
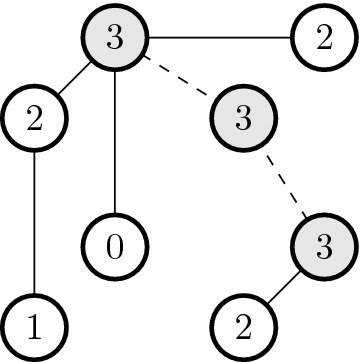
Graph in a cube. Vertices represent genotypes; filled edges connect sequences at one mutation distance; dashed edges connect sequences of high fitness in a RSE instance; and filled vertices represent peaks

Following Example 1, we represent the corresponding graph in Fig. 4. Note that if a vertex has multiple neighbors with maximal fitness, one may choose one arbitrarily. Edges in $E_1$ (resp. $E_2$) are represented by solid (resp. dashed) lines.

***Connectedness of the graph***

We now prove that *G* is connected, and therefore its first Betti number ($b_0$, the number of connected components) is one. First, note that any vertex is connected to a peak. Indeed, from any vertex, by following the path of fittest mutations, we can go to a peak by edges in $E_1$. Therefore, we only need to prove that all peaks are connected.

By contradiction, assume that there are $K_1, \ldots , K_r$ connected components of *G*. Note that in each component there might be multiple peaks. We will define a special path of single mutations. Consider the “usual” sequence graph, formally the hypercube $G_S = (\{0, 1\}^d, E_{d_1})$, where $E_{d_1}$ containing all edges connecting sequences at Hamming distance one. Take the path $P_*$ that connects two peaks in different components and has the highest minimum value, i.e.,Without loss of generality, assume that $P_*$ connects $v_1 \in K_1$ and $v_2 \in K_2$. We will show that the two connected components $K_1$ and $K_2$ are in fact connected, which is a contradiction.

Denote $v_m$ the vertex in $P_*$ that achieves the minimum fitness. Divide $P_*$ into the path before and after $v_m$, formally: $P_* = P_*^1 v_m P_*^2$. Our first observation is the following: all vertices in $P_*^1$ are in $K_1$, i.e., $V(P_*^1) \subseteq K_1$, and similarly all vertices in $P_*^2$ are in $K_2$. Indeed, if it was not the case, consider $v' \in P_*^1 \cap K_1^c$. Since $v' \not \in K_1$, by following the fittest mutation, it is connected to a peak $v_2'$ which is not in $K_1$. Consider a new path $P_*'$ that goes from $v_1$ to $v'$ and then to $v_2'$. Note that the minimum fitness value in $P_*'$ is higher than the one in $P_*$ and $P_*'$ also connects two different connected components, which is a contradiction. Therefore, $V(P_*^1) \subseteq K_1$. Similarly, we get that $V(P_*^2) \subseteq K_2$. Having identified this property of $P_*$ and $v_m$, we can construct a path in our graph of interest *G*, instead of $G_S$.

Denote $u_{m}^1 \in K_1$ the vertex in $K_1 \cap P_*^1$ closest to $v_m$, similarly denote $u_{m}^2$ the vertex in $K_2 \cap P_*^2$ closest to $v_m$. First notice that $\{u_{m}^1, u_{m}^2\} \in E_2$, i.e., there is a reciprocal sign epistatic interaction between vertices with high fitness. Indeed, if this were not the case, we could connect them through another mutation that does not involve $v_m$ and create a path $P_*'$ with a higher minimum value, which is a contradiction. Since $\{u_{m}^1, u_{m}^2\} \in E_2$, i.e., are connected in *G*, all we need to do to construct our desired path connecting $K_1$ and $K_2$ is showing that $u_{m}^1$ is connected to a peak in $K_1$ and similarly $u_{m}^2$ is connected to a peak in $K_2$.

Since $u_{m}^1 \in K_1$, we can follow the fittest mutation path until a peak $u_1 \in K_1$ (similarly for $u_{m}^2$ to a peak $u_2 \in K_2$). Consider the paths $Q_*^1$ and $Q_*^2$, where  and . By definition of $E_1$, we have that $Q_*^1, Q_*^2 \subseteq E_1$. Finally, the path connecting two different peaks (assumed to be disconnected) is $Q_* = Q_*^1 Q_*^2$.

Indeed, note that $Q_*$ is a path in $E = E_1 \cup E_2$ since $Q_*^1 \subset E_1$, $\{u_{m}^1, u_{m}^2\} \in E_2$ and $Q_*^2 \subset E_2$. Therefore, the peaks $u_1 \in K_1$ and $u_2 \in K_2$ are connected. But this is a contradiction because $K_1$ and $K_2$ were two different connected components. This concludes the proof that *G* is connected.

***Definition of a function***

Consider the function $f: K \rightarrow \mathbb {R}$ given by the following.

- For all $$v \in V$$,
  $$\begin{aligned} f(v) = - W(v) \,. \end{aligned}$$
- For all $$e = {u, v} \in E_1$$,
  $$\begin{aligned} f(e) = \frac{f(u) + f(v)}{2} \,. \end{aligned}$$
- For all $$e = {u, v} \in E_2$$,
  $$\begin{aligned} f(e) = C \,, \end{aligned}$$
  where $$C > \max \{ |W(v) |: v \in V\}$$.

**Fig. 5:**
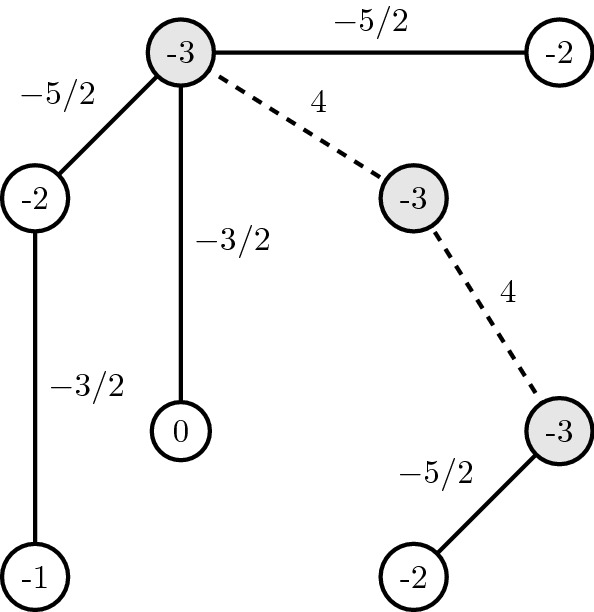
Function in the graph. Vertices represent genotypes; filled edges connect sequences at one mutation distance; dashed edges connect sequences of high fitness in a RSE instance; edges and vertices are labelled with the value of the defined function; and filled vertices represent peaks

Following Example 1, we represent the corresponding function in Fig. 5. Note that edges have values and we have chosen $C = 4$.

***Application of Morse inequality***

By Theorem 2, we have that

$$\begin{aligned} m_1 - m_0 \ge b_1 - b_0 \,. \end{aligned}$$

Since *M* is connected, we have that $b_0 = 1$. By definition of Betti numbers, and since *M* is connected, $b_1 \ge 0$ (see Remark 1). The number of critical vertices is $m_0$ and the number of critical edges is $m_1$. By construction, the only critical vertices are peaks. Indeed, a vertex is critical if all edges containing it have greater value. Since all single mutations and edges in $E_2$ have greater values than the peak value, all peaks are critical. Moreover, non-peak values have a beneficial mutation and therefore an edge in $E_1$ with a greater value. Therefore, non-peak vertices are not critical. On the other hand, the only critical edges are those in $E_2$, i.e., edges that represent reciprocal sign epistatic interaction, since these edges are the only ones whose endpoints have both smaller values. Therefore,

$$\begin{aligned} \#\text { RSE instances }\ge \# \text { peaks } - 1 \,. \end{aligned}$$

$$\square $$

## Discussion

We have shown that the multipeaked fitness landscape necessarily has no fewer pairwise reciprocal sign epistatic interactions than the number of fitness peaks minus one. This extends the result of Poelwijk et al. (2011) stating that the reciprocal sign epistasis is a necessary condition for multiple peaks. Additionally, our study showcases the application of discrete Morse theory to fitness landscapes. As our paper was in review, a different way to prove the same result was posted by Chenette et al. (preprint) providing extra confidence in this result. The main difference in our approaches is that we based our proof on Morse theory, while the proof by Chenette et al., relies on explicit constructions of fitness landscapes. In this work, the authors also prove that multipeaked fitness landscapes must have at least as many reciprocal sign epistatic interactions as the number of fitness peaks minus one. They also studied how many instances of RSE can exist when there is only one fitness peak. Moreover, having upper and lower bounds on the number of instances of RSE given a certain number of fitness peaks, they studied if all numbers in between the bounds can be realized by some fitness landscape.

More empirically characterized fitness landscapes are becoming available, driven by high throughput mutational scan studies. One straightforward way to analyze them is to determine the number of fitness peaks in the landscape. Our results may allow biologists to deduce the minimum number of reciprocal sign epistatic interactions in their data based on the number of observed fitness peaks.

As discussed in Poelwijk et al. (2011), in the general case, reciprocal sign epistasis is not a sufficient condition for multiple peaks. Similarly, we do not show how to estimate the number of peaks from the number of RSE instances. The task of deducing global properties of the landscape from its local properties was accomplished in Crona et al. (2013): they showed that reciprocal sign epistasis is a sufficient condition for the existence of multiple peaks if there is no sign epistasis in any other pair of loci.

**Fig. 6:**
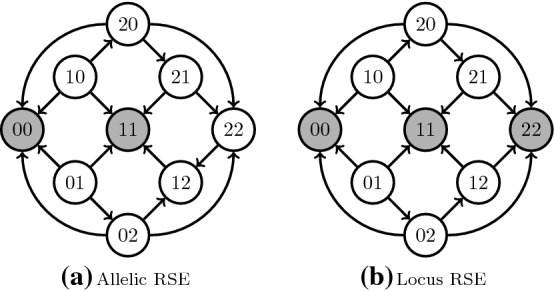
Reciprocal sign epistasis with more than two alleles. Vertices represent genotypes; their label is their genotype sequence; filled vertices represent peaks; and arrows represent fitness increments

In our proof, we considered bi-allelic genotypes, which may not reflect the biology of DNA or protein sequences. The application of our theory to genotypes with more than two alleles depends on how epistasis is defined for such genotypes. Epistatic interactions may be found between alleles at different loci, which may lead to instances where some allele states between two sites are in an epistatic interaction, while two different allele states at the same sites may not show any epistasis. Alternatively, epistasis may be defined when there is an epistatic interaction between any alleles at two different sites. Such epistasis may be called *allelic* and *locus* epistasis, respectively. See Fig. 6 for an illustration. Our proof is immediately generalizable without modifications for fitness landscapes determined by allelic epistasis but not necessarily to landscapes determined by the locus epistasis. We believe that allelic epistasis is biologically more realistic than locus epistasis and, therefore, our proof is relevant for fitness landscapes determined by DNA or protein sequences.

For our proof we assumed that the fitnesses of all genotypes are different, while empirically some fitness landscapes may be “neutral” in that many genotypes may have the same fitness, as has been observed in some empirical landscapes (Aguilar-Rodríguez et al. 2017; Schaper et al. 2012). However, the difference between fitnesses in our model can be arbitrarily small, many orders of magnitude smaller than the experimental error. Therefore, the difference in fitnesses we introduce in our proof does not impact the application of our results to empirically characterized fitness landscapes. Generally speaking, reciprocal sign epistasis is value-agnostic, in that any magnitude of the effect is taken into account as long as the sign of the effect changes. Therefore, the small variance in the values of fitnesses that we introduced does not influence the detection of sign epistasis in real data.

The complication of deducing the global properties of fitness landscapes from the local properties of epistasis between specific sites arises due to the multidimensionality of the fitness landscape: local peaks formed by a pairwise epistatic interaction can be bypassed through a different dimension. Therefore, the condition formulated in terms of the pairwise epistatic interaction cannot be sufficient. One needs to know the full fitness landscape: to deduce that the fitness landscape has multiple peaks, one has to know that there is no sign epistasis in any other pairwise interaction (Crona et al. 2013).

For a quantitative result converse to ours, we anticipate that higher-order epistatic interactions have to be considered, which leads to the requirement of full information about the fitness landscape. We expect that this result can be obtained with a suitable definition of the higher-order epistasis. Such a result could be useful, for example, to study the empirical fitness landscapes if the number of mutations under consideration is small enough to make an almost complete description of the landscape feasible.

## Data Availability

Data sharing does not apply to this article as no datasets were generated or analyzed during the current study.
