# Correction to: Relation Between the Number of Peaks and the Number of Reciprocal Sign Epistatic Interactions

**DOI:** 10.1007/s11538-022-01118-z

**Published:** 2023-01-21

**Authors:** Raimundo Saona, Fyodor A. Kondrashov, Ksenia A. Khudiakova

**Affiliations:** Institute of Science and Technology Austria, Am Campus 1, 3400 Klosterneuburg, Lower Austria Austria

**Correction to: Bull Math Biol** 10.1007/s11538-022-01029-z

The original version of the article unfortunately contained mistakes. It has been corrected in this correction.

## Corrections

There was an incorrect citation of a theorem from Morse Theory (Forman 1998, Corollary 3.6, page 107). Some assumptions were missing. This mistake is corrected as follows.

1. Add the necessary assumptions in the theorem.
2. Complete the proof that uses this theorem (by showing that the assumptions hold in the context of our proof).

## Morse Function Assumption

Theorem 2, Section 4, page 3 of 12, is a reference to a result from Morse Theory given by Forman (1998, Corollary 3.6, page 107). The result was wrongly cited since there is a missing assumption about the properties of the functions that the theorem applies to. This does not invalidate the conclusions of Theorem 2 since the function we construct satisfies these missing properties.

The necessary changes are as follows.

1. Theorem 2, Section 4, page 4 of 12, adds the assumption that the function $f : V \cup E \rightarrow \mathbb {R}$ needs to be a Morse function.
2. Section 4.1, “Necessary definitions”, page 5 of 12, introduces the definition of Morse function for graphs. Formally, the following definition.

### Definition 1

(Morse function) Let $G = (V, E)$ be a graph. The function $f:V \cup E \rightarrow \mathbb {R}$ is a Morse function if the following conditions hold.

- All vertices have at most one edge with lower or equal value. Formally, for all $v \in V$,
  $$\begin{aligned} \vert \{ u \in V : e = \{ u, v \} \in E, \quad f(e) \le f(v) \} \vert \le 1 \,. \end{aligned}$$
- All edges have at most one vertex with lower or equal value. Formally, for all $e = \{ u, v \} \in E$,
  $$\begin{aligned} \vert \{ u \in V : \exists v \in V \quad e = \{u, v\}, \quad f(e) \ge f(v) \} \vert \le 1 \,. \end{aligned}$$

3. Section 4.2, “Proof”, page 5 of 12, adds a step in the proof consisting of proving that the function defined is a Morse function.
4. Section 4.2, “Proof”, page 5 of 12, proves that the function defined is a Morse function as follows.
  By the definition of Morse functions, we have to show a property for each vertex and each edge. For each vertex *v*, we need to show that there is at most one edge connected to *v* whose value is lower or equal to *f*(*v*). Indeed this is the case since edges in $E_2$ have the highest value possible and, by construction, there is at most one edge in $E_1$ connecting *v* with its fittest mutation. All other edges connected to *v* come from a vertex with strictly lower fitness. Therefore, if *v* is not a peak, there is exactly one edge connected to *v* with a lower value. If *v* is a peak, all edges connected to *v* have strictly higher values than *f*(*v*).
  For each edge *e*, we need to show that at most one of its vertices has a value larger or equal to *f*(*e*), but not both. Indeed this is the case for $e \in E_1$, since the value of *f*(*e*) is the average of the value of its vertices. Edges in $E_2$ have the highest possible value, so this property also holds. In conclusion, the function *f* is indeed a Morse function.
